# Granular stockpile volume dataset

**DOI:** 10.1016/j.dib.2026.112631

**Published:** 2026-02-27

**Authors:** Faezeh Jafari, Sattar Dorafshan

**Affiliations:** Department of Civil Engineering, Advanced Transportation Infrastructure Center, University of North Dakota, Grand Forks, ND 58202, USA

**Keywords:** Flight mission, UAS imagery, 3D point cloud, Annotation, Stockpile volume estimation

## Abstract

Unmanned Aerial Systems (UAS) applications are growing for vision-based volume measurement to enhance accuracy, efficiency, and automation. Despite the growing applications of UAS, no comprehensive dataset is currently available for researchers to determine the effects of visual data collection parameters such as camera angles, image overlaps, and flight patterns, on the outcomes. These outcomes consist of but are not limited to the number of images, the density of point clouds, and the quality of 3D models. This study introduces an annotated UAS dataset to allow researchers and practitioners to use vision-based UAS data for accurate measurement of granular stockpiles. Data were collected from stockpiles with irregular shapes in Grand Forks, ND, USA using UAS. The dataset includes 1521 images captured under varying weather conditions, stockpile sizes, camera angles, flight patterns, flight heights, and image overlaps. This study investigated 47 stockpiles across two distinct sites, including sand and gravel materials. Using Pix4D photogrammetry, 3D models were generated, with individual stockpile volumes ranging from 51 m³ to 3000 m³. Data was collected during multiple surveys; however, stockpiles were not individually tracked across time, so the dataset should be regarded as cross-sectional rather than strictly longitudinal. Stockpile volumes in one of the sites changed overtime during the data collection. The dataset was enriched with annotated 3D points identifying not only stockpiles, but irrelevant objects, such as trees, vehicles, and roads. The point clouds generated from these models were annotated in PLY and XYZ formats, creating a unique 3D point dataset with corresponding 2D images. This dataset is well-suited for the development of autonomous detection and measurements of objects using 3D deep learning models for object detection.

Specifications Table

**Table:** 

Subject	Engineering (Construction, Mining, Aerospace, Computer Science, Agriculture)
Specific subject area	Civil Engineering.
Data format	Raw 2D images and annotated 3D point cloud.
Type of data	RGB image (JPG), 3D points clouds in XYZ (.xyz) and PLY (.ply) formats
Data collection	DJI Phantom 4 Pro was employed to collect images of regular and irregular objects. The UAS is compatible with Pix4D, allowing for autonomous path planning. Pix4D was utilized to conduct autonomous flights and generate 3D models by setting flight parameters such as altitude, overlapping, and flight mission. The software also facilitated image stitching, 3D model construction, and the creation of annotated datasets.
Data source location	North Dakota Department of Transportation Stockpile site, Grand Forks, ND. University of North Dakota Stockpiles site: Tech Accelerator, Address: 4201 James Ray Dr, Grand Forks, ND 58202.
Data accessibility	Repository name: Granular stockpile Identification and Measurement using a UAS-based Multidimensional dataset. Data identification number: Direct URL to data: https://figshare.com/s/5481148a8b761b1f5aa3
Related research article	Jafari, F., & Dorafshan, S. (2025). Vision based stockpile inventory measurement using uncrewed aerial systems. *Ain Shams Engineering Journal, 16*(2), 103251.

## 1 Value of the Data

• Captured using diverse flight patterns, camera angles, and point cloud density, the dataset fills a critical gap by providing detailed path planning parameters not typically available in existing datasets.
• Enables evaluation and identification of optimal flight patterns for more effective data collection.
• Supports volume measurement, density analysis, and stockpile characterization, making it useful for sectors like construction, transportation, mining, and manufacturing.
• Facilitates the development and benchmarking deep learning models for 2D image classification, 3D modeling, and point cloud-based object detection.
• Offers a practical and cost-effective solution for researchers without access to expensive LiDAR systems or stockpile sites, while still enabling accurate 3D analysis.
• Allows augmentation of data attributes (e.g., size, density, brightness), providing a flexible foundation for training, validating, and refining data-driven models.

## 2 Background

Pix4D is a widely used commercial software that provides path planning for data collection, applied in various civil engineering fields such as construction monitoring, measurement, and inspection [1]. Among the various path patterns, circular and double grid paths are the most common. Increasing overlap or adjusting the camera angle in a circular pattern can directly affect the quality of the data, as well as the completeness of the area and the algorithm's ability to match features between images [2]. Optimizing path planning improves model quality, ensures full coverage of required inspections, and reduces analysis time [3]. Previous studies have shown that different path planning approaches at varying flight heights can impact 3D model quality, stockpile volume measurement, data collection time, and processing time [4]. A dataset with adjusted flight parameters, such as camera angle, overlap, and flight pattern, when extracting data from the same area, can provide UAS pilots using Pix4D with more information before starting data collection. Autonomous path planning is widely used for stockpile monitoring in both industry and research. Data are typically collected by selecting an appropriate path, after which stockpiles are analyzed in terms of volume, 3D model quality, and density [5]. Lasers, UAS LiDAR, and UAS imagery have been investigated in previous research for volume estimation. Researchers typically test their algorithms using one type of data, such as 3D point clouds or images, without employing autonomous object detection. This research introduced a new dataset to support researchers aiming to evaluate the impact of flight parameters on path planning. Furthermore, based on a limited point cloud dataset over a stockpile’s objects, two large stockpiles were monitored with different flight patterns, and point clouds were generated for various objects. This dataset can be widely applied for various purposes, particularly in stockpile monitoring, path planning, and the application of deep learning for 3D point cloud classification [[1], [2], [3], [4]]. Creating 3D datasets using various devices such as UAVs, robotic-based systems, handheld cameras, and LiDAR has been explored in recent studies. However, to meet diverse user needs, further expansion and development of such datasets are still necessary [[6], [7]].

## 3 Data Description

The dataset contained 2D images collected from two stockpile sites, offering valuable data for studying stockpile characteristics such as volume, shape, and density. The component features annotated point cloud data summarizing the objects captured in the dataset, enabling advanced applications such as 3D modeling and object detection. Table 1 presents the flight parameters and data organization for the UAS dataset collected in 2022, 2023 and 2024 from two stockpile site locations. Each flight is identified by a unique Flight ID and associated with a specific type of mission, including double grid, polygon, 2D grid, and circle flight patterns. The table summarizes key flight characteristics, including the Ground Sampling Distance (GSD), point cloud density (points per cubic meter), camera angle during the flight, and the number of images captured. The GSD values range from 0.52 cm to 3.68 cm, indicating varying levels of spatial resolution based on the mission design and site requirements. The point cloud density also varies widely, from 229 points/m³ to over 24,000 points/m³, reflecting differences in image overlap and flight altitude. Overall, the dataset reflects a diverse set of flight patterns, resolutions, and data collection strategies, making it suitable for various applications, including 3D modeling, volume estimation, and infrastructure assessment. In Pix4D autonomous missions, a double grid pattern refers to two sets of parallel flight lines flown in perpendicular directions, forming a cross-hatched grid over the survey area. This ensures high overlap and consistent coverage, making it suitable for Ortho mosaics and volumetric analysis. The camera angle parameter in Pix4D defines the tilt of the UAV camera relative to nadir (vertical). A value of 90° corresponds to a nadir (straight-down) orientation, while smaller values indicate increasingly oblique image capture. For example, in our dataset, double grid missions were flown at 90° (nadir) or 80° (slightly oblique). For circle missions, Pix4D also allows images to be triggered at specified angular intervals along the orbit (e.g., every 10° around the stockpile). In our dataset, this 10° value in Table 1 corresponds to the image acquisition interval, not the gimbal tilt. A detailed description of each part is provided below:

• Images of the dataset are captured using the UAS, with flight heights ranging from 35 to 300 feet. The stockpiles, varying in color and material, are stored in separate subfolders. The main variables considered in this section include flight height, flight pattern, stockpile material, and stockpile size.
• Annotated 3D Point Cloud: This folder contains annotated 3D point cloud data for objects such as trees, cars, roads, and stockpiles, captured from various flights. The data is provided in PLY and XYZ formats containing point coordinates and RGB information. An 80% image overlap was used for all flights. In addition to extracting the object from the flight from the stockpile in Table 1 with mentioned flight ID, 3D objects from other flights in this project with overlaps higher than 80% were also included and stored in additional point cloud folders (EPC folder). Since the primary goal of this part was to generate a 3D dataset and enhance diversity, the corresponding 2D images were excluded due to space limitations.

**Table 1:** Dataset file organization for stockpile’s dataset

	Flight ID	Type of mission	Flight parameter			Number of images	3D point cloud
			GSD (cm)	Density (per m3)	Camera angle		
NDDOT	NDDOT-1	Double grid	0.73	8835	90	478	PLY and XYZ
	NDDOT-2	Double grid	1.6	866	90	121	PLY and XYZ
	NDDOT-3	Polygon	0.73	7249.74	90	181	PLY and XYZ
	NDDOT-4	Polygon	1.60	917.21	90	143	PLY and XYZ
	NDDOT-5	Polygon	2.33	229.27	90	118	PLY and XYZ
	NDDOT-6	Double grid	1.19	1016.75	80	98	PLY and XYZ
	NDDOT-7	Circle	1.42	997.45	10	36	PLY and XYZ
	NDDOT-8	Circle	3.68	60.67	10	68	PLY and XYZ
UND	UND-1	Circle	2.06	431.92	10	32	PLY and XYZ
	UND-2	Double grid	0.52	24304.1	80	70	PLY and XYZ
	UND-3	2D gird	1.65	705.76	90	30	PLY and XYZ
	UND-4	Polygon	1.68	694.46	90	17	PLY and XYZ
	UND-5	Double grid	1.69	768	90	64	PLY and XYZ
	UND-6	Circle	0.78	5395.62	10	32	PLY and XYZ
	UND-7	Circle	0.89	4285.53	10	33	PLY and XYZ

Table 2 summarizes the organization of the UAS-based dataset collected in previous recent years. The dataset includes information on flight identification (Flight ID), total surveyed area, stockpile volumes, stockpile areas, flight times, and flight dates. In all flights, except for NDDOT 7 and NDDOT 8, there were multiple stockpiles on site. The maximum, minimum, and average values for volume and area were extracted and reported in this table. The dataset covers flights conducted by both the North Dakota Department of Transportation (NDDOT) and the University of North Dakota (UND). The size of the surveyed areas varies significantly, ranging from 2,500 m² to 366,000 m². Similarly, the stockpiles captured in the flights exhibit a wide range of volumes and areas, reflecting diverse site conditions. The flight durations range from 1 minute to 25 minutes depending on the project scale and survey requirements. Additionally, the dataset reflects diversity in stockpile sizes, time of data collection (including both morning and evening flights across different months), and flight patterns, providing a robust and varied dataset for applications such as volume estimation, stockpile monitoring, and model development.

**Table 2:** Flight summary: UAS-based 3D stockpile modeling and volume estimation

	Flight ID	Total Area in 3D model (m^2^)	Stockpile volumes (m^3^)			Stockpile Areas (m^2^)			Flight time (min)	Flight date
			Min	Mean	Max	Min	Mean	Max		
										
NDDOT	NDDOT-1	22294	47	1121	2082	175	1555	2394	25	October 6, ‏6:44:39 PM
	NDDOT-2	38495	22	911	2268	125	547	1145	8	October 6, ‏6:30:52 PM
	NDDOT-3	19729	47	1121	2082	175	1555	2394	8	August 9, 2022, ‏5:12:00 PM
	NDDOT-4	19729	23	988	2561	1390	606	102	9	October 6, ‏6:23:04 PM
	NDDOT-5	148318	224	1520	2530	58	2408	8400	8	October 6, 2022, ‏5:57:32 PM
	NDDOT-6	3790	23	303	584	51	326	601	5	August 22, 2022, ‏5:53:00 PM
	NDDOT-7	13816	1125	1125	1125	1677	1677	1677	2	August 12, 2022, ‏1:17:00 AM
	NDDOT-8	366000	1140	1140	1140	1700	1700	1700	3	October 3, 2022, ‏9:17:00 AM
UND	UND-1	5206	4	43	86	75	104	134	1	August 9, 2022, ‏5:11:00 PM
	UND-2	2500	4	43	86	75	104	134	3	August 9, 2022, ‏5:12:00 PM
	UND-3	18932	15	48	76	145	104	63	5	May 3, 2023, ‏4:24:55 PM
	UND-4	12989	17	61	98	69	108	138	1.62	Friday, May 5, ‏3:26:38 PM
	UND-5	24563	15	48	76	145	104	63	10	May 3, 2023, ‏4:36:09 PM
	UND-6	5000	39	63	86	75	104	134	2	August 9, 2022, ‏5:11:00 PM
	UND-7	4285.53	39	63	86	75	104	134	2	August 9, 2022, ‏5:12:00 PM

## 4 Experimental Design, Materials and Methods

To validate the usefulness of the dataset, a deep learning-based point cloud classification model was developed to distinguish stockpiles from surrounding terrain and irrelevant objects. The model was trained using the annotated 3D point clouds generated from UAS imagery of stockpiles collected through Pix4D [4]. The classification 1DCNN model achieved an average volume difference of less than 5.5% compared to Pix4D-generated reference volumes. It significantly reduced manual processing time by automating the detection and volume estimation of stockpiles from point cloud data. This approach offers a cost-effective alternative to LiDAR-based methods for construction, mining, and transportation sectors, making it a valuable tool for stockpile monitoring, especially where access to LiDAR or high-cost scanning equipment is limited. This baseline experiment demonstrated how stockpile dataset could be directly applied in machine learning–based stockpile monitoring. The results serve as a reference point for future researchers to evaluate different methods and highlighted potential limitations (e.g., sensitivity to flight parameters. To further validate the dataset, we compared volume estimates against UAS LiDAR–derived point clouds. Models derived from the datasets were within ±6% of LiDAR-based measurements [4].

### 4.1 UAS data collection

**Fig. 1:**
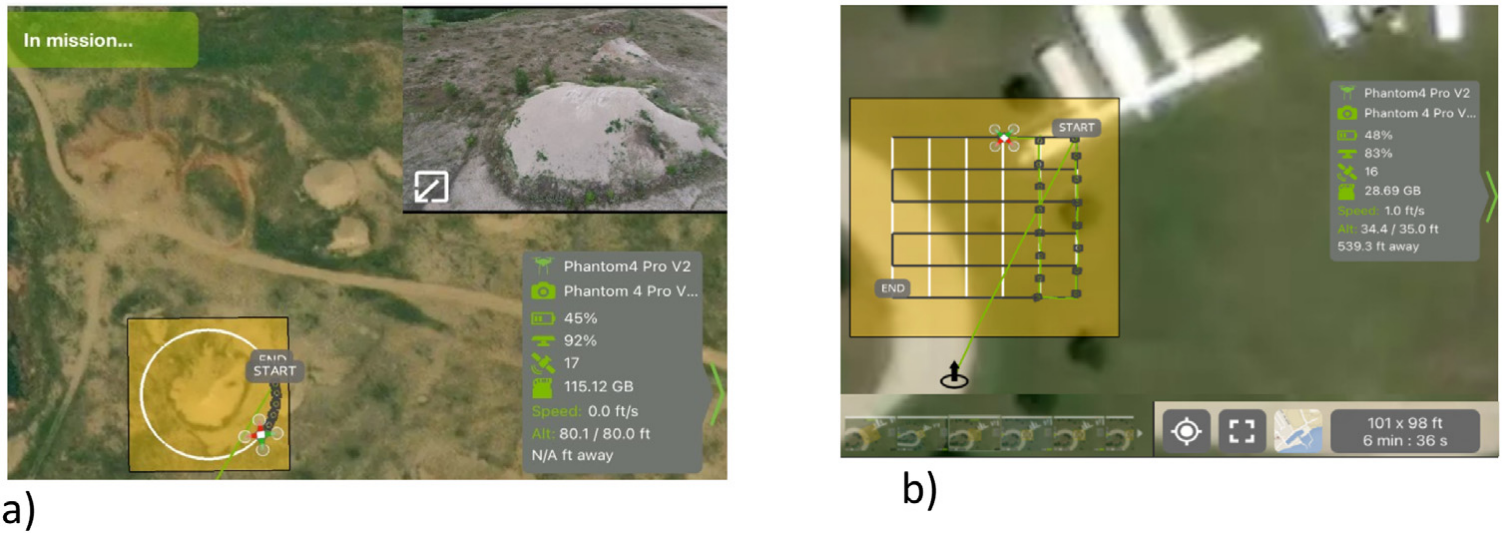
Autonomous flight pattern for the a) circle pattern and b) double grid pattern.

Pix4D is a photogrammetry and automated flight planning software used to complete flights and generate 3D models. In manual mode, the user controls UAS movement via remote control (RC), whereas in autonomous mode, flights are fully monitored by the autopilot. Figures 1a and 1b depict the circle and double grid patterns most common flight pattern used for data collection, respectively (Fig. 1).

**Fig. 2:**
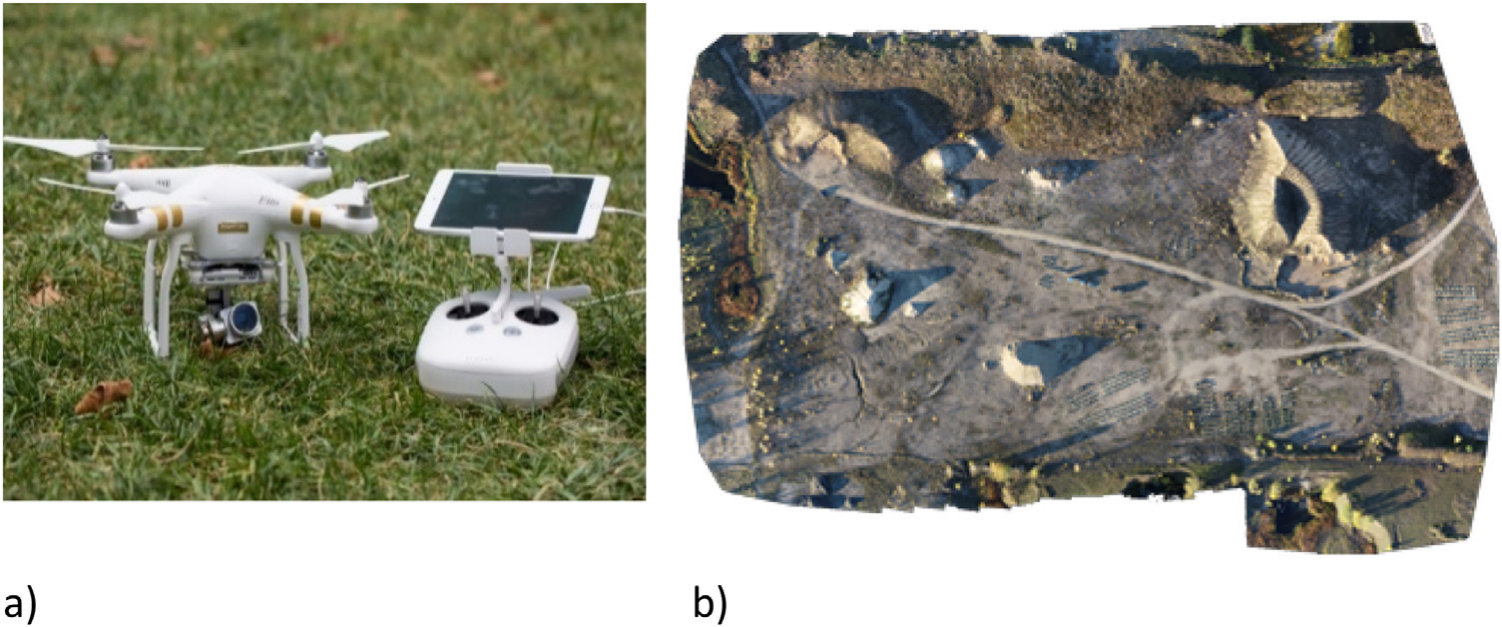
a) Phantom 4 DJI used for data collection, b) 3D models of the NDDOT site in Grand Forks.

Path planning for data collection was autonomously managed through Pix4D and Phantom, which enables image stitching and three-dimensional model construction. The software allows the user to define flight parameters such as flight pattern, height, overlap, and camera angle. Multiple flights were conducted to capture images of stockpiles. For both sections of the dataset, Phantom was used to perform the flights (Fig. 2a) and Pix4D was used to annotate the data in 3D point cloud format. Flight parameters, including the type of flight pattern, camera angle, and image overlap, were adjusted before each flight. Multiple flight missions with different flight heights were employed to capture images from two stockpiles sites in Grand Forks. Fig. 2b shows the stockpiles at the NDDOT site, captured with a polygonal flight pattern at a height of 300 ft.

### 4.2 UAS Data annotation

**Fig. 3:**
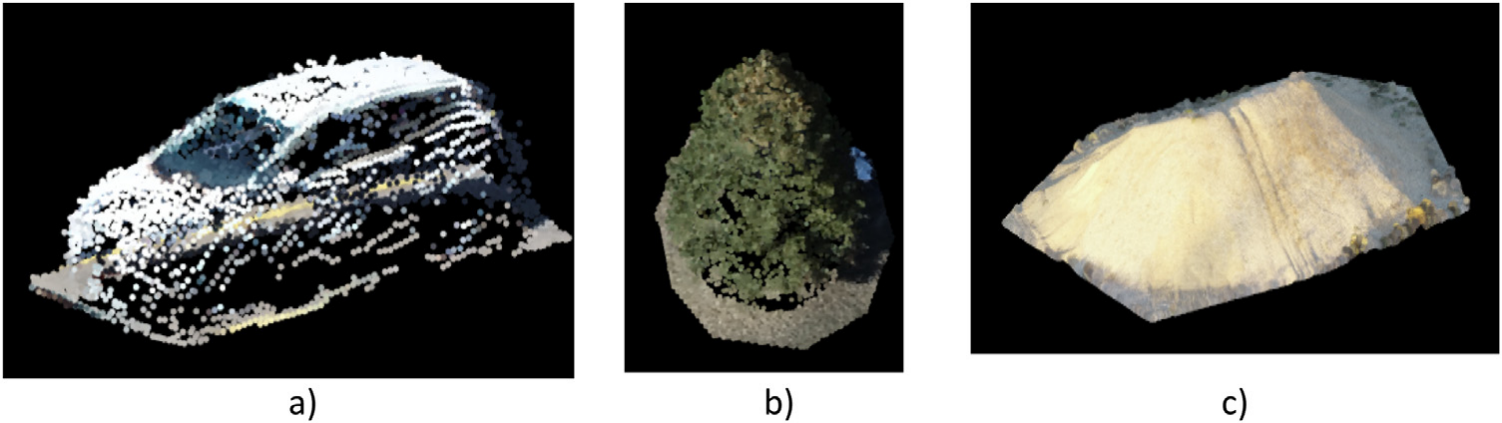
3D annotated file in XYZ format, a) Car, b) Tree, c) Stockpile.

**Fig. 4:**
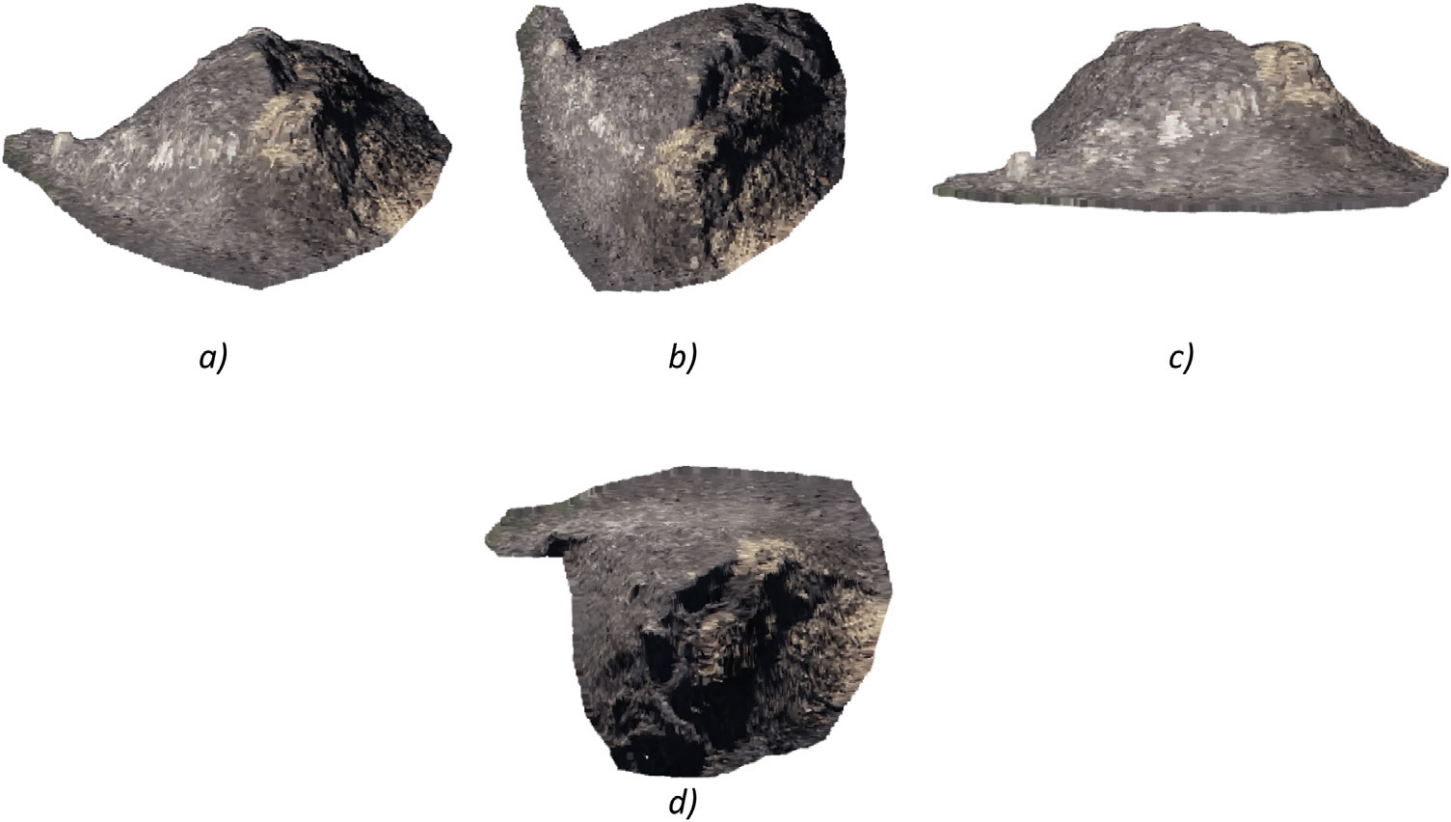
Generate multiple viewpoints of the same stockpile point cloud, a) Isometric view (45,45), b) top view, c) Front view (0,0)), d) Bottom view (0, -90).

The dataset presented in this article is unique, as it is the only stockpile dataset with 3D annotated stockpiles available at the time of writing. Additionally, other 3D objects, such as cars (Fig. 3a), trees (Fig. 3b), and stockpiles (Fig. 3c), were also annotated in XYZ and PLY formats in this study. Unlike previous studies that primarily used LiDAR for 3D point classification, this dataset includes 3D point cloud annotations extracted from 2D images. The annotation process was carried out using Pix4D [8] software, without any post-processing, to ensure clean data that separates objects from the ground—where the base may be considered a secondary object in deep learning algorithms. This process resulted in user-defined annotations [4], where the boundaries of the objects are outlined within the 3D point cloud data. These boundaries were then used for training the classification 1DCNN model. Since the process involves user manual input, the accuracy of the boundaries depends on the user's judgment, rather than being automatically generated by the software. Figs. 4a, b, c, and d show different views of one stockpile as main object of this dataset.

These annotations were then used for training the 1DCNN deep learning model. This approach allowed for the segmentation of stockpiles from other objects, which can then be used for further analysis, such as volume calculation, without the need for manual boundary clicking during the model's application.

To ensure data quality and annotation reliability, each labeled object was cross validated by user through visual inspection several times and comparison against raw orthophotos and 3D models. This manual validation process was essential to confirm the accuracy of object boundaries and to minimize misclassification. No pre-processing algorithms were applied after annotation to preserve the integrity of the raw dataset, which is critical for training deep learning models that must learn to differentiate objects from their natural surroundings. In total, the dataset includes 47 stockpile point clouds and 23 annotated non-stockpile objects. The table presents the number of instances, object types, and prediction accuracy. Using the 1D-CNN model, the classification accuracy for distinguishing stockpiles from other objects reached 95.5% (Table 3).

**Table 3:** A 3D annotated stockpile dataset

Class	Number of Instances	Type of Object	Accuracy Rate (%)
Stockpile	47	Stockpile (gravel, sand, aggregate)	95
Other Objects	23	Tree, car, ground, parking lot, railroad, wood, grass	96

After creating this dataset, the authors used a 1D CNN to classify stockpiles from other objects. We employed user-defined pseudo-ground truth (pseudo-GT) annotations to segment and classify objects such as stockpiles, trees, and cars. In Pix4D software, the author manually clicked to define the boundaries of these objects based on their judgment. This process resulted in user-defined annotations, where the boundaries of the objects were outlined within the 3D point cloud data. These boundaries were then used for training the classification 1D CNN model. Since the process involved manual input, the accuracy of the boundaries depended on the user’s judgment rather than being automatically generated by the software. These annotations were then used as a form of pseudo-GT for training the deep learning model (1D CNN). This approach allowed for the segmentation of stockpiles from other objects, which was subsequently used for further analysis, such as volume calculation, without the need for manual boundary clicking during the model’s application [4].

## Limitations

• A key limitation of data collection using Unmanned Aerial Systems (UAS) is compliance with FAA Part 107 regulations, which prohibit flying in controlled airspace, over people, and near heavy traffic without special waivers.
• Based on Pix4D's limitation in selecting flight height less than 35 ft, autonomous path planning has been used to collect most 2D images with a minimum allowable flight height of 35 ft with 0.6 GSD values. Decreasing flight height led to more 2D image quality.

## Ethics Statement

This research was supported by the North Dakota Department of Transportation (NDDOT). The authors declare no conflicts of interest. This research does not involve studies with human participants or animals. The authors confirm that the work complies with the ethical requirements for publication in *Data in Brief*, as outlined by Elsevier.

## Credit Author Statement

**Faezeh Jafari:** Conceptualization; Methodology; Software; Data curation; Investigation; Writing – original draft; Visualization. Sattar Dorafshan: Supervision; Writing – review & editing; Project administration.

## Data Availability

GIMUN: A Multidimensional UAS Dataset for Granular Stockpile Identification and Volume Measurement (Original data).
